# An overview of the advanced data collection techniques in the environmental exposure unit (EEU)

**DOI:** 10.1186/1710-1492-10-S2-A1

**Published:** 2014-12-18

**Authors:** Daniel E Adams, Barnaby Hobsbawn, Terry JB Walker, Lisa M Steacy, Anne K Ellis

**Affiliations:** 1 Allergy Research Unit, Kingston General Hospital, Ontario, Canada; 2 Department of Medicine, Queen’s University, Kingston, Ontario, Canada

## Background

Capturing symptom data for large studies (≥60 participants) in ≤30 minutes is hard to achieve using manual data entry without substantial costs and resources. This challenge is highlighted if the data are needed to make real-time clinical decisions. The 140 participant capacity of the Environmental Exposure Unit (EEU) requires an advanced method to process symptom data.

## Methods

Advanced scanning technologies are used with a customized two-step quality assurance data collection process. Optical Mark Recognition (OMR) and Optical Character Recognition (OCR) capture data from paper symptom diary cards into the EEU’s clinical data management system (CDMS). A template is configured to read the static diary card format and assign zones where the specific diary card data are located. The user configures field requirements within the zones to validate data captured. Cards that do not meet a predefined confidence level for any particular zone will be flagged for a quality check. The quality checking process involves one user visually confirming all data captured and a second user inputting all values from the card to ensure accuracy. Invalid data are rejected from the batch and returned to the participant for correction. Corrected cards are scanned again and all valid data are transferred into the CDMS.

## Results

Capturing data using the advanced scanning system allows a team of 3 to process 120 symptom diary cards containing 9 symptoms and 3 peak nasal inspiratory flow (PNIF) scores, with 99.9% accuracy in <15 minutes. In comparison, manual data entry would require a team of 8 to achieve similar results.

## Conclusions

For large studies with short assessment periods, the scanning system utilized in the EEU is significantly more efficient in all aspects of data acquisition than manual entry. This ability to accommodate large studies in an accurate, efficient manner leads to an ideal setting for the conduct of time-sensitive clinical trials.

